# Integrating European contexts and needs into WHO guiding principles on online mental health content for young people: five recommendations

**DOI:** 10.1007/s00787-024-02566-9

**Published:** 2024-08-29

**Authors:** Helena Ludwig-Walz, Martin Bujard, Jonas Fegert, Jörg M. Fegert

**Affiliations:** 1 Federal Institute for Population Research (BiB), Wiesbaden, Germany; 2 Institute of Medical Psychology, Medical Faculty, University Heidelberg, Heidelberg, Germany; 3 Institute of Information Systems and Marketing (IISM), Karlsruhe Institute of Technology (KIT), Karlsruhe, Germany; 4 Department for Child and Adolescent Psychiatry and Psychotherapy, University Medical Center, Competence Domain Mental Health Prevention, Ulm, Germany; 5 German Centre for Mental Health (DZPG) partner site Mannheim, Heidelberg, Ulm, Germany

The World Health Organization (WHO) has published guiding principles on online mental health content for young people [1], recognizing the transformative role of online technologies in providing mental health support. This article aims to integrate the key principles of the WHO report into European contexts and needs, to enhance the efficacy and relevance of online mental health services for young people.

## Need for European youth mental health action

Mental health conditions greatly contribute to the disease burden among young people in Europe [2]. Young people in Europe face a multitude of challenges, including academic and peer pressures, the impact of online social networks (OSN; including social media and messaging services) and external crises like the climate crisis, COVID-19 aftermath, and the Ukraine war. OSN enhance global connectivity but also contribute to emotionalization and polarized discourse [3]. The COVID-19 pandemic from 2020 to 2022 affected approximately 105 million pupils and students in Europe and accelerated a mental health crisis, resulting in a rise in depression and anxiety symptoms [4, 5]. The lasting effects of COVID-19 on youth mental health and the overstretched psychiatric and psychological care systems are still evident, making it crucial to ensure that young people have access to the support and resources needed to navigate these challenges successfully [6]. This is also an ethical obligation, since the youth generation greatly contributed to the safety of older people during the pandemic [7].

## Online mental health services for young people

The digital transformation has altered how young people seek and consume information, while the mental health care system, particularly in Europe, has lagged behind. Traditional services, such as face-to-face counselling and telephone helplines, are overwhelmed and struggling to adapt to new communication habits. The COVID-19 pandemic has further integrated online tools into daily life.

Technological support in youth mental health shows promise, particularly for anxiety and depression [8], though study results remain heterogeneous. We are still in the early stages of designing and developing online interventions and platforms, facing challenges such as data privacy concerns, the fact that not all platforms provide evidence-based or qualified support, the limited ability of online services to offer immediate assistance in crisis situations, ethical challenges, especially in child protection cases and trauma care, and the difficulty standardized online programs may have in addressing the individual needs and specific issues of users.

Ground-breaking developments in the use of artificial intelligence (AI), including generative AI, are both revolutionary (e.g., assessment of mental states and even forecasting psychiatric relapses) and concerning. Chatbots offer real-time support, but AI in mental health care poses challenges like model biases, underscoring the need for stringent regulatory oversight.

## WHO 2023 report: guiding principles for youth mental health online content

The WHO’s 2023 report on online mental health content for youth defines 10 guiding principles from a virtual roundtable, aiming to enhance and protect mental health [1]:

1. Emotional relevance.
2. Practical strategies.
3. Cognitive fit.
4. Language.
5. Inclusivity and diversity.
6. Lived experience.
7. Visual engagement.
8. Evidence-based clarity.
9. Accessibility.
10. Human rights alignment.

## Online mental health for European contexts and needs

As the WHO report offers a global perspective, designing and developing online mental health services for European youth requires considering the socio-cultural, technological, and regulatory contexts of the EU region.

### Goal conflicts between evidence-based clarity and practical strategies

For some of the WHO’s guiding principles, there is currently a conflict of goals, leading to sub-optimal outcomes (see Table 1). Traditional mental health services often refer to evidence-based guidelines, with access to these services is regulated as a benefit under social law. However, these services are increasingly losing contact with the young target group. Contrarily, online mental health services are flexible, fast, and target-oriented, but they often lack certified quality and integration into a stepped-care health service model. The core challenge lies in bridging the gap between validated quality and accessibility to young people, as the institutional logics and actors are very different.

**Table 1:** Overview of key conflict areas between Traditional and Online Mental Health Services

	Traditional Mental Health Services	Online Mental Health Services
Advantage for guiding principle	Evidence-based Clarity	Practical Strategies
Certification	Validated certification	Limited or non-standardized certification
Reaching the young target group	Low	High
Provider Competence	30–70 years (after PhD)	15–29 years (digital natives)
Principles of decisions	Scientific boards	Start-ups with input from few experts
Speed of processes	Slow	Fast
Status of service	Standardized	Flexible
Duration of mental health service	Short, medium and long	Short

### Reflecting and researching online realities and socio-cultural contexts

European youth typically gain early access to advanced technology, especially in economically advanced regions where smartphones, tablets, and computers are ubiquitous. Many children own mobile phones by the age of 10 and often have their own laptop as early teenagers, with this trend becoming increasingly common at even younger ages. OSN play a pervasive role in shaping their social interactions and perceptions of mental health. Despite the transparency obligations mandated by the EU Digital Services Act (DSA), OSN have not achieved the required level of transparency. Researching the effects of their hyper-personalization, which varies across socio-economic milieus and demographics, remains challenging. Techniques like TWONs (Digital Twins of OSN) and A/B testing can clarify the impact of algorithms and content engineering on children and adolescents, particularly regarding mental health. Independent institutions should conduct these tests to identify problematic platform mechanisms and algorithmic biases, thus eliminating reliance on voluntary data donations and enabling researchers, civil society, and regulators to address these issues directly with platform providers.

Europe’s diverse socio-cultural landscape, with its multitude of languages, cultural practices, and social norms, influences how young people perceive and engage with mental health resources. While mental health is openly discussed and accepted in some regions, stigma and misconceptions persist in others. Despite these differences, Europe is generally more open to acknowledging and seeking help for mental health issues compared to other regions. Also, socio-economic disparities across Europe affect access to technology and mental health services. Some areas boast high online penetration and robust mental health support systems, while others may lack the necessary infrastructure.

This early and extensive interaction with technology highlights the need for online mental health services that align with the practices of digitally native users and bridges gaps by offering accessible, culturally sensitive services for all young Europeans.

### Involving families and youth in online mental health solutions

Digital literacy among families in Europe varies due to factors such as education, socio-economic status, and location. Northern European countries are among the frontrunners in providing digital literacy training, ensuring that both parents and children can navigate online environments effectively. Other regions require targeted interventions to ensure all parents can support their children’s online and mental health needs. A supportive family environment is crucial for integrating mental health strategies into daily life, thereby enhancing the effectiveness of online interventions.

Children and adolescents, with their deep understanding of their mental health needs and extensive experience with digital technologies, are key contributors to the design and development of online mental health services. Co-designing these services with young people leads to innovative solutions that are effective and resonant. Although still new and cautiously implemented, initiatives like the WHO Young Researchers Forum highlight the potential of involving youth in enhancing mental health care quality [9]. Therefore, participatory technology development methods, including stakeholder-based requirement engineering and the evaluation of the IT-artifacts with its potential users, help ensure that these services are developed according to the stakeholders’ needs.

### Need for a regulatory framework

The surge in digital mental health interventions, often driven by independent initiatives like ‘Krisenchat’^1^ (Crisis Chat) in Germany, highlights the urgent need for a cohesive regulatory framework. These essential services are largely disconnected from formal healthcare systems. In Europe, there is a gap between traditional healthcare settings, where access to preventive measures is regulated, and the digital space as a novel healthcare setting, where such integration is lacking. Addressing this requires targeted recommendations for a stepped-care approach to mental health interventions for children and young people at the European level, ensuring standardized certification and oversight to guarantee safety, effectiveness, and ethical standards.

Currently, the regulation of medical devices in Europe is handled at the country level, resulting in a relatively heterogeneous landscape. For example, the German Medical Devices Act has established complex regulations comparable to drug authorization. As a result, no app has been approved as a locatable medical device for young people in Germany, in contrast to adult psychiatric use. Additionally, the rise in popularity of generative AI chatbots, which pose challenges such as hallucinations and misinformation, underscores the need for a trusted health ecosystem to combat disinformation and promote literacy [10]. AI models can inherit biases from training data, leading to discriminatory outcomes that stigmatize certain groups. To ensure these technologies are safe and effective, strict regulatory and monitoring mechanisms – including regular audits and the development of ethical guidelines – are essential to maintain transparency and inclusivity in AI-driven mental health solutions.

## Five core recommendations

1. *European Context Matters*: Online mental health services must be tailored to the specific socio-cultural, technological, and regulatory contexts of Europe to be effective and relevant for youth.
2. *Balancing Quality and Access*: It is necessary to bridge the gap between the evidence-based quality of traditional mental health services and the accessibility of online platforms to better reach young people.
3. *Involvement is Key*: Enhancing digital literacy among families and involving young people in the co-design of services can significantly improve the effectiveness of online mental health interventions.
4. *Addressing AI Challenges*: The rise of AI in mental health care necessitates stringent oversight to prevent biases and misinformation, ensuring that AI-driven solutions are transparent and inclusive.
5. *Regulatory Framework Needed*: The rapid growth of digital mental health tools underscores the urgent need for a cohesive regulatory framework to ensure safety, effectiveness, and ethical standards.

## Data Availability

No datasets were generated or analysed during the current study.

## Footnotes

1 ‘Krisenchat’ is a free crisis counselling service in Germany that provides immediate psychological support via chat for children, adolescents, and young adults.
